# A programmable DNA origami nanospring that reveals force-induced adjacent binding of myosin VI heads

**DOI:** 10.1038/ncomms13715

**Published:** 2016-12-12

**Authors:** M. Iwaki, S. F. Wickham, K. Ikezaki, T. Yanagida, W. M. Shih

**Affiliations:** 1 Quantitative Biology Center, RIKEN, OLABB, 6-2-3, Furuedai, Suita, Osaka 5650874, Japan; 2 Graduate School of Frontier Biosciences, Osaka University, Suita, Osaka 5650871, Japan; 3 Department of Cancer Biology, Dana-Farber Cancer Institute, Boston, Massachusetts 02115, USA; 4 Department of Biological Chemistry and Molecular Pharmacology, Harvard Medical School, Boston, Massachusetts 02115, USA; 5 Wyss Institute for Biologically Inspired Engineering, Harvard University, Boston, Massachusetts 02115, USA; 6 School of Frontier Sciences, The University of Tokyo, Kashiwa, Chiba 2778561, Japan; 7 Center for Information and Neural Networks, NICT, Suita, Osaka 5650874, Japan

## Abstract

Mechanosensitive biological nanomachines such as motor proteins and ion channels regulate diverse cellular behaviour. Combined optical trapping with single-molecule fluorescence imaging provides a powerful methodology to clearly characterize the mechanoresponse, structural dynamics and stability of such nanomachines. However, this system requires complicated experimental geometry, preparation and optics, and is limited by low data-acquisition efficiency. Here we develop a programmable DNA origami nanospring that overcomes these issues. We apply our nanospring to human myosin VI, a mechanosensory motor protein, and demonstrate nanometre-precision single-molecule fluorescence imaging of the individual motor domains (heads) under force. We observe force-induced transitions of myosin VI heads from non-adjacent to adjacent binding, which correspond to adapted roles for low-load and high-load transport, respectively. Our technique extends single-molecule studies under force and clarifies the effect of force on biological processes.

## Abstract (web summary)

Characterizing the mechanical response of molecular motors involves the use of methods such as optical trapping to apply force. Here the authors develop a DNA origami nanospring to apply progressive force to human myosin VI, and discover that it adopts different stepping modes when subjected to low load or high load.

Mechanical forces regulate the function of numerous mechanosensory protein molecules including motor proteins^1^, ion channels^2^, cytoskeletal filaments^3^ and cadherins^4^. These molecules are involved in essential cellular behaviours such as cell growth, differentiation, shape formation and cell death^5^. Optical trapping (OT) provides a powerful methodology to explore the molecular mechanics and mechanoresponse of these biomolecules at the single-molecule level^1^,^3^,^4^,^6^,^7^,^8^. OT functions by applying force to nano-sized molecules via optically trapped particles (generally, 0.2∼1 μm in diameter). Although displacements with nanometre accuracy can be acquired using OT, it is difficult to determine intramolecular or subdomain motion within the molecule. Instead, single-molecule fluorescence (SMF) imaging provides a powerful method to monitor structural dynamics within biomolecules by attaching fluorophores to the subdomains^9^,^10^,^11^. Therefore, simultaneous OT and SMF has been developed^12^,^13^,^14^,^15^, but it is technically difficult to apply due to a number of factors caused by large-scale devices that use a high-power infrared beam to trap microspheres including low throughput (that is, only one biomolecule can be analysed at a time) and complicated experimental geometry, preparation and optics that minimize dye photobleaching induced by the infrared trapping beam. Furthermore, the use of fluorescent quantum dots (QDs) or gold nanoparticles (GNPs) to circumvent dye bleaching issues is problematic due to their attraction to the trapping beam's focus point.

To overcome these issues, we developed a nanoscale force-application device using three-dimensional DNA origami^16^,^17^. Our device is a programmable DNA nanostructure with a coil shape (nanospring) and we applied it to study the mechano-response of the actin-based processive motor protein, myosin VI. Myosin VI is a ubiquitously expressed mechanosensitive dimeric motor protein, which is thought to act as an anchor to maintain the structural integrity of many cellular structures such as stereocilia^18^ and the Golgi apparatus^19^. In addition, myosin VI is a vesicle transporter that moves cargo along cytoskeletal actin filaments coupled to the hydrolysis of ATP^20^,^21^. It has been proposed that mechanical forces cause myosin VI to switch between these roles of anchor and transporter by slowing its ATP hydrolysis cycle at the strongly bound state with actin^20^,^22^, as well as causing a transition from hand-over-hand to inchworm-like stepping^23^. In inchworm-like stepping, step sizes of the motor domains (or heads) are 44 nm, suggesting the rear head becomes the lead head and forms adjacent binding state (that is, adjacent motor domains close together), which could better resist detachment due to an even distribution of load between the two heads, and therefore could be seen as an adaptation for anchor function. Therefore, Nishikawa *et al*.^23^ predicted that high load should lead to a higher fraction of adjacent binding states observed. To test this hypothesis, SMF imaging of myosin heads in the presence of load is required; however, combined OT and SMF imaging was difficult to implement for the study of molecular machines; therefore, no direct test has yet been reported.

An ideal nanospring for myosin VI would exhibit a relatively small spring constant at the stall force of the motor under investigation, which in the case of myosin VI, is reported to be ∼2 pN^20^,^22^; otherwise, only very few steps could be tracked close to the stall force. In addition, the nanospring should not be overly long (that is, >>1 μm) to avoid the requirement of extended motor run lengths that stretch the nanospring; this is problematic, as the average run length before spontaneous detachment of myosin VI is ∼700 nm (ref. 24). Our programmable nanospring can fully satisfy the desired characteristics unlike natural elastic components such as double-stranded DNA (dsDNA). In addition, SMF imaging of the nanospring–biomolecule complex is superior to simultaneous OT and SMF imaging, in that we can visualize over ten complexes at a time using conventional total internal reflection fluorescence microscopy (TIRFM), and QDs and GNPs are also available. Furthermore, a nanospring can control the number and species of biomolecules in the complex and is compatible with other useful methods such as high-speed atomic force microscopy (AFM)^25^, dark-field microscopy and electron microscopy without complicated optics or geometries.

Here we demonstrate SMF imaging of single- and double-colour QDs attached to the heads of myosin VI dimers tagged with our nanospring and also the tug-of-war between myosin VI and myosin V (opposite directional motors). We find force and ADP synergistically trigger transitions from hand-over-hand to inchworm-like movement of myosin VI and stalling in the adjacent binding state, thus providing insights into the mechanism of the physiological dual function of myosin VI as a vesicle transporter and anchor.

## Results

### Design and mechanical properties of nanospring

**Figure 1:**
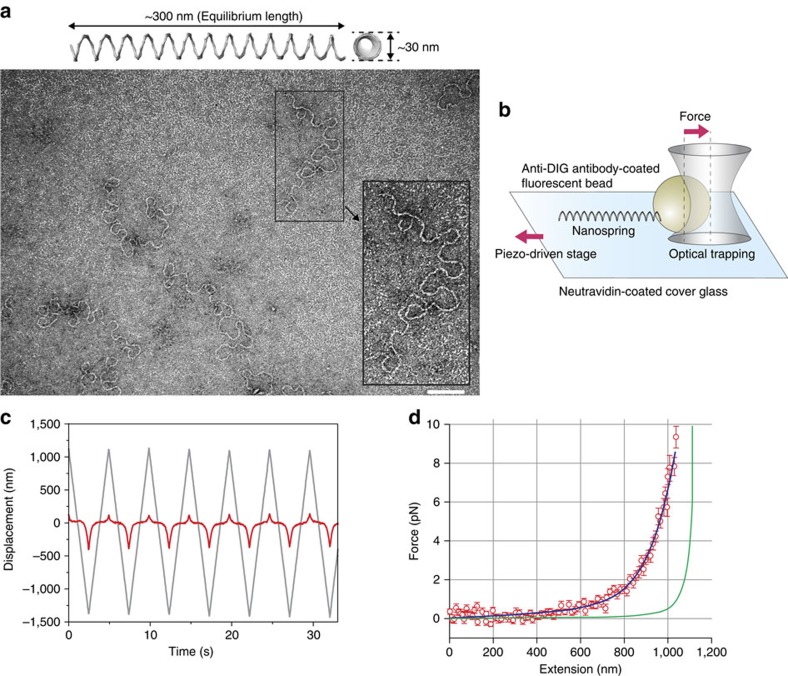
Programmable DNA origami nanospring and the force-extension curve. (**a**) Upper: the three-dimensional structure of a two-helix bundle (2HB) nanospring produced by CanDo^43^, which predicted a coil structure (13 turns, ∼30 nm in diameter and∼300 nm in equilibrium length). Lower: a transmission electron microscopy image of a nanospring folded in 14 mM MgCl_2_. Inset: expanded view of a typical nanospring. The shape was flexible enough to measure single myosin VI force but also showed expected loops. Scale bar, 100 nm. (**b**) Experimental setup for the OT assay. A 200 nm fluorescent bead tagged with a nanospring (bead-NS) was optically captured and the cover glass was then moved by a piezo actuator. (**c**) Trajectory of the bead (red line) and fix point of the 2HB nanospring on the cover glass (grey line). See also Supplementary Movie 1. (**d**) Averaged force-extension curve for the 2HB nanospring. *N*=19. Error bars represent s.d. Blue line, fitted curve by a seventh-order polynomial. Green curve indicates a theoretical curve for a single dsDNA assuming a modified Marko–Siggia worm-like chain model^26^.

Our nanospring consists of a two-helix bundle programmed with negative superhelical strain to form a coil structure (Fig. 1a and Supplementary Figs 1 and 2). To construct the nanospring, 7,308-nucleotide single-stranded DNA (ssDNA; scaffold) and >150 species of short (<50 nucleotide) ssDNA (staples; Supplementary Data) were self-assembled by hybridization under rapid heating followed by gradual cooling. To attach specific protein molecules to the ends of the nanospring, extra ssDNA handles were projected out from each end. The length or number of bundles and turns of the nanospring is programmable (Supplementary Fig. 2b), resulting in control of the spring constant.

To estimate the force-extension curve, we applied force to the nanospring using OT (Fig. 1b and Supplementary Fig. 3). We attached a fluorescent polystyrene bead at the end of the nanospring and optically trapped it. The other end was fixed to a glass slide and moved back and forth (Fig. 1c and Supplementary Movie 1) to obtain a force-extension curve (Fig. 1d). The curve was reversible between stretching and relaxing phases (Supplementary Fig. 3f), and the change in the slope (spring constant) was more gradual than that of dsDNA over a range of several pN. We also confirmed the force-extension curve was not affected by the rate of stretching (Supplementary Fig. 3g). Our two-helix bundle nanospring exhibited a spring constant of 0.012±0.002 pN nm^−1^ (mean±s.d., *N*=19) and a length of 841 nm at 2 pN of force (contour length of 1.1 μm), which were estimated from the force-extension fit curve (Fig. 1d). For myosin VI, which takes a 36 nm step^20^, these properties increases the force by 0.43 pN, which is a moderate change in force and useful for facilitating better resolution of the force–velocity relation near the stall force. In contrast, a dsDNA spring of the same length would be far less sensitive. At 2 pN of force, a 1.1 μm contour length of dsDNA would exhibit a spring constant of ∼0.09 pN nm^−1^, or roughly an order of magnitude larger^26^. To achieve a comparably compliant spring would require a dsDNA of length ∼5 μm (ref. 26), which would then impose a limitation of analysis to run lengths of that magnitude, which are very rare (∼0.08%)^24^.

### Nanospring dynamics driven by a single myosin VI

**Figure 2:**
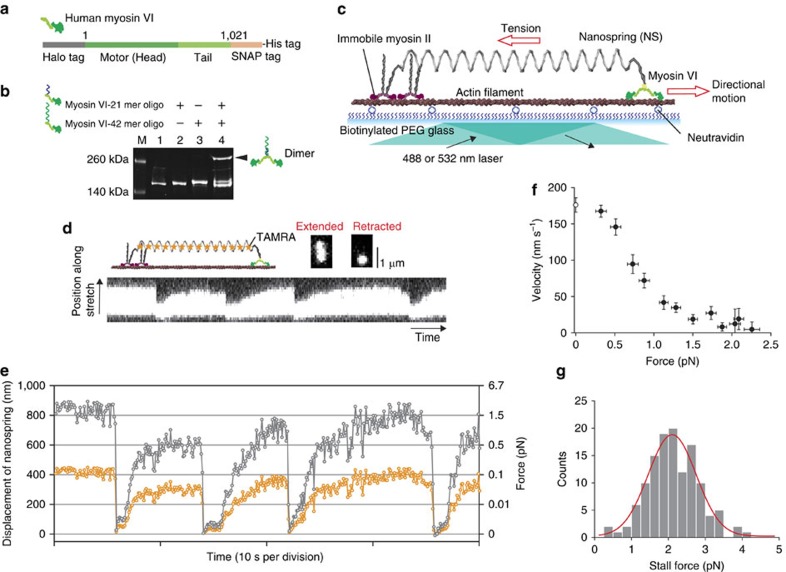
**Nanospring**–**motor protein complex to measure single-molecule dynamics under load.** (**a**) Monomeric myosin VI constructs. (**b**) A gel-shift assay confirmed the labelling of oligos and DNA-induced dimerization. M, marker; lane 1, myosin VI monomer; lane 2, myosin VI monomer labelled with 21 mer oligo (myosin VI-21 mer oligo); lane 3, myosin VI monomer labelled with 42 mer oligo (myosin VI-42 mer oligo); lane 4, myosin VI-21 mer oligo mixed with myosin VI-42 mer oligo. The black arrowhead indicates a dimer fraction. (**c**) Myosin VI tethered to a two-helix bundle (2HB) nanospring moves unidirectionally along actin against the load of the nanospring. (**d**) Visualization of the stretch/compression dynamics of the nanospring by myosin VI at 2 mM ATP+100 μM ADP. The kymograph shows repetitive stretching and compressing of the TAMRA-labelled nanospring. (**e**) Trajectory of the centre position of the fluorescence image of the nanospring (orange circles). The displacement was multiplied by 2, to measure the spring extension from which the force was calculated (grey circles). (**f**) Averaged force–velocity curve at 2 mM ATP+100 μM ADP. Error bars represent s.e.m. Velocities at 0 pN force were measured by tracking myosin VI-NS in the absence of immobile myosin II (open circle, *N*=28). (**g**) A stall force histogram. Stall over 2 s were included in the analysis. The red line marks a a Gaussian function fit (2.1±0.6 pN (mean±s.d.)).

Myosin VI is composed of an amino-terminal motor domain (head) and tail domain. The tail consists of four subdomains: the proximal, medial and distal tails and the carboxy-terminal cargo-binding domain^27^. The proximal and medial tails can potentially act as a lever arm that contributes to force generation and the cargo-binding domain regulates dimerization^24^,^27^. Myosin VI is thought to function as a dimer in cells^24^ and adaptor proteins at the cargo-binding domain are necessary for regulating the equilibrium between the monomeric and dimeric forms^28^. In the present study, to make a stable myosin VI dimer, we engineered a myosin VI monomer fusion protein in which the cargo-binding domain was replaced with SNAP-tag, whereas preserving all other myosin components (Fig. 2a). SNAP-tag can be efficiently conjugated to an oligonucleotide (oligo) that serves as an artificial dimerization domain^29^. The myosin VI monomer was also fused to HaloTag at the N terminus of the head to enable fluorophore labelling. We covalently labelled 21-mer or 42-mer oligos to our myosin VI monomers and confirmed >90% labelling efficiency by a gel-shift assay (Fig. 2b). As the 42-mer oligo contains a complementary sequence to the 21-mer oligo, the mixture showed a dimer fraction due to hybridization. The remaining sequence in the 42-mer oligo retains a domain that captures the ssDNA handle in the nanospring (capture domain).

To observe the stepping dynamics of myosin VI under tension, we designed a myosin VI–nanospring complex (myosinVI-NS; Fig. 2c), where one end of the nanospring was attached to a DNA-coupled myosin VI dimer via the nanospring capture domain on the myosin VI 42-mer oligo, whereas the other end of the nanospring was attached to an immobilized myosin II dimer, which was chemically inactivated by *N*-ethylmaleimide and labelled with ssDNA. Immobile myosin II strongly binds to actin filaments in the presence of ATP and plays a role in fixing myosinVI-NS on the actin filament.

We used TIRFM to observe the dynamics of a fluorescently labelled nanospring driven by a single myosin VI (Fig. 2d). Thirteen unlabelled staples were replaced with fluorescently labelled staples (5′-end was labelled with TAMRA; Supplementary Data) that were homogeneously positioned in the nanospring. As the equilibrium length of the nanospring without tension is ∼300 nm (that is, close to half the wavelength of the fluorescence emission), the fluorescence image is seen as a diffraction-limited circular spot (Fig. 2d, compressed). When myosin VI stretches the nanospring against tension, the shape becomes elliptical (Fig. 2d stretched and Supplementary Movie 2). By fitting the images to a two-dimensional (2D) Gaussian distribution^30^, we were able to determine the centre position and length of the nanospring to an accuracy of 14±2 nm (mean±s.d., *N*=11) when extended longer than 500 nm total spring length (Fig. 2e and Supplementary Fig. 4). Typically, myosin VI stretched the nanospring ∼800 nm and stalled. In this state, it eventually detached from the actin and the nanospring returned to the compressed state after the release of tension. This sequence of events was repeatedly observed. We quantified the tension using a force-extension curve (Fig. 1d). From this curve, we estimated the stall force to be 2.1±0.6 pN (mean±s.d., *N*=126; Fig. 2f,g), which is in agreement with previous force measurements using conventional optical tweezers^20^,^22^.

### Tug-of-war between myosin V and myosin VI via nanospring

**Figure 3:**
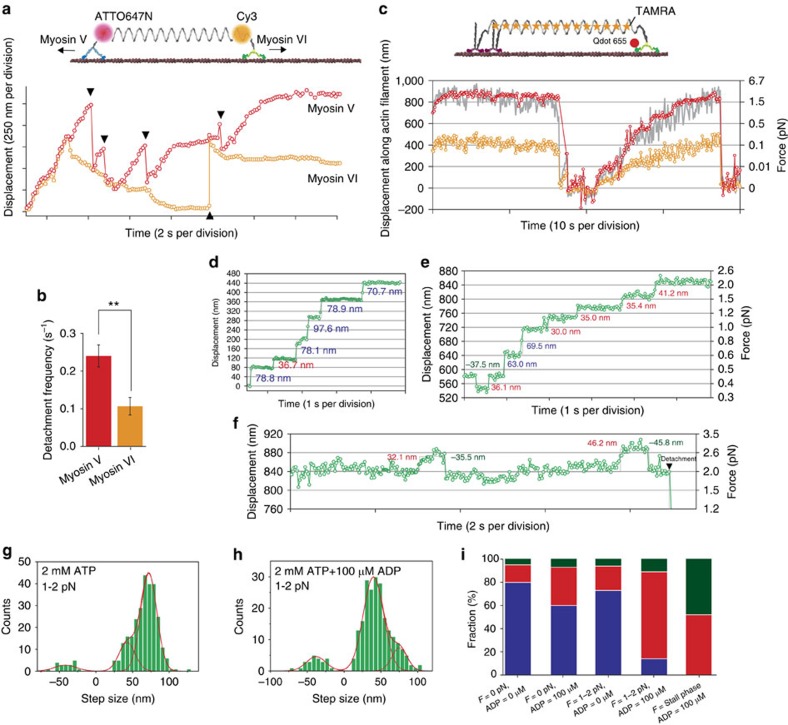
Dual colour imaging of single-molecule tug-of-war and nanoimaging of myosin VI head domain under load. (**a**) Typical trajectory for dual colour imaging of tug-of-war between myosin V and VI at 2 mM ATP+100 μM ADP. Red circles, the ATTO647N position, which indicates myosin V motion; orange circles, the Cy3 position, which indicates myosin VI motion. Black triangles indicate detachment events. (**b**) Detachment frequency during tug-of-war for myosin V (red) and myosin VI (orange). Error bars indicate s.e.m. *N*=47 myosin-NS complexes, **P<0.01 (two-sided *t*-test). (**c**) Trajectories of a QD (red circles) attached to the head of a myosin VI, the centre position of a TAMRA nanospring (orange circles) and two times the displacement of the centre position of the TAMRA nanospring (grey line) at 2 mM ATP+100 μM ADP. Recording rate, 10 Hz. (**d**–**f**) Trajectories at 2 mM ATP and 0 pN (**d**), 2 mM ATP+100 μM ADP and 0.4-2 pN (**e**), and stall force (**f**). Recording rate, 30 Hz. The red Gaussian fits in **g** represent step sizes at 2 mM ATP and 1–2 pN load (−41.8±15, 42.5±11 and 72.1±11 nm) and in **h** at 2 mM ATP+100 μM ADP and 1–2 pN load (−38.0±13, 40.0±13 and 75.3±10 nm). Error indicates s.d. (**i**) The distribution of large (blue), short (red) and backward (dark green) steps at different loads and ADP concentrations estimated from the Gaussian fits.

Myosin VI has been proposed to function as an anchor and a transporter, whereas myosin V has been proposed to function exclusively as a transporter^31^. Our DNA nanospring afforded us the opportunity for direct comparison of their motile properties by a tug-of-war between single myosin V and VI (Fig. 3a and Supplementary Movie 3). As expected, myosin V and VI (indicated by ATTO647N and Cy3 fluorescence, respectively) moved processively along the same actin filament but in opposite directions^32^,^33^. The nanospring was stretched further and further until one myosin or the other detached from the actin filament. We observed myosin VI to detach less than half as frequently as myosin V (Fig. 3b), consistent with the dual role proposed for myosin VI.

### Detection of head motion in myosin VI dimer under load

Next we observed the stepping dynamics of QD-labelled myosin VI in the absence and presence of the nanospring using TIRFM at ∼2 nm and 30 Hz spatio-temporal resolution^23^,^30^. In the absence of the nanospring and at saturating ATP concentration (2 mM), our DNA-coupled myosin VI dimer moved processively along actin filaments (Supplementary Fig. 5 and Supplementary Movie 4). The step-size distribution for the myosin head (Supplementary Fig. 5b) was fit to a three Gaussian function with peaks at 75 nm (large forward steps), 43 nm (short forward steps) and −40 nm (backward steps), consistent with previous reports^23^,^34^. The distribution of dwell times just before the forward steps (Supplementary Fig. 5c) were well fit to the formula *tk*^2^exp(−*kt*) if assuming two rate-limiting steps having the same rate constant, *k*. We obtained *k*=4.8 s^−1^, which is also consistent with previous reports^20^,^22^,^23^. In conclusion, we confirmed the fusion of SNAP-tag and DNA to induce dimerization did not alter the stepping dynamics or coordination between heads. Further, we also examined the stepping dynamics of myosinVI-NS (Supplementary Fig. 6a,b) and confirmed the labelling of nanospring also did not alter the motion.

When a myosinVI-NS was linked with an actin filament via immobile myosin II, myosin VI exhibited processive walking, stalling and detachment coupled with the extension and retraction of the nanospring (Fig. 3c and Supplementary Movie 5). The QD-myosin VI trajectory (red circles in Fig. 3c) was in agreement with the nanospring displacement (grey line in Fig. 3c) and stall forces calculated from QD displacements (Supplementary Fig. 6c) were consistent with previous reports^20^,^22^ and those estimated from nanospring displacements (Fig. 2g). Further, we could clearly measure the step sizes of individual myosin VI heads at 30 Hz recording rate (Fig. 3d–f). In the absence of ADP at 1–2 pN load (Fig. 3g), the stepping behaviour was similar to that in the absence of the nanospring (that is, 0 pN load) and large forward steps (75 nm) were the most frequent step type. However, when we added 100 μM ADP (physiological nucleotide concentration), the most frequent step type changed to short forward steps (43 nm) and the frequency of backward steps (−40 nm) slightly increased (Fig. 3h). Figure 3i shows a summary of the distribution of large forward, short forward and backward steps as a function of load and ADP concentration (ATP concentration was held constant at 2 mM). Large forward steps were dominant both with and without 100 μM ADP at no load (Fig. 3d), which is in agreement with a previous report^23^. We found a similar distribution at 1–2 pN load in the absence of ADP. However, the distribution shifted to short forward steps at 1–2 pN load and 100 μM ADP (Fig. 3e), and at stall force (∼2 pN) only short forward and backward steps were observed (Fig. 3f). Thus, our results show a transition in the myosin VI step size distribution in response to ADP and load.

### Dynamics of both heads of a myosin VI dimer under load

**Figure 4:**
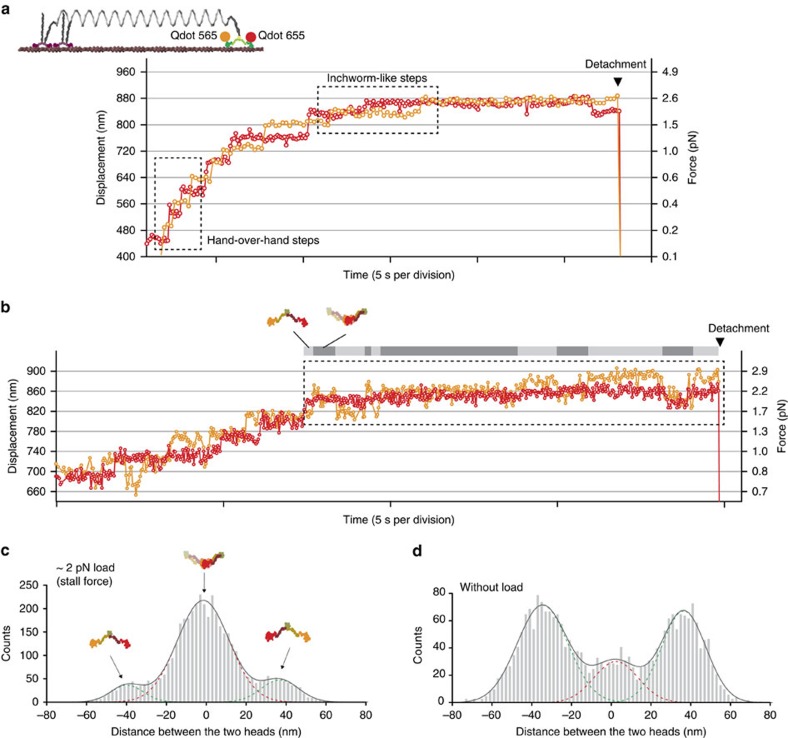
Simultaneous observation of both heads under load. (**a**) Typical trajectory of QDs attached to both heads showing hand-over-hand steps and inchworm-like steps (dashed-line box). (**b**) Typical trajectory of QDs attached to both heads in the stall state (dashed-lined box). The grey and light grey bars indicate the binding state of myosin VI onto actin (grey, adjacent binding states; light grey, distant binding states). Recording rate, 10 Hz for (**a**) and 30 Hz for (**b**), respectively. Nucleotide condition, 2 mM ATP+100 μM ADP. The arrowhead shows detachment from actin. (**c**,**d**) Distance between heads at stalled state (**c**) and at unloaded state (**d**). The distribution fit to three Gaussian functions (solid and broken lines) with peaks at 37±9.3 nm, −1.5±13 nm and −39±8 nm for **c**, and 36±11 nm, 2.4±11 nm and −34±12 nm for **d**. The peak around 0 nm is interpreted as a convolution of positive and negative distances for the adjacent binding state. Error indicates s.d.

To fully clarify the stepping behaviour, we monitored both heads of a myosin VI dimer by attaching different colour QDs to them. Simultaneous nanometric observation^23^,^34^,^35^ of the heads allowed us to determine the coordination and distance between them. We confirmed that the large forward steps we observed were caused by a hand-over-hand movement (Fig. 4a, dashed-line box) and the short forward steps were caused by inchworm-like movement^23^,^34^ (Fig. 4a, dashed-line box); therefore, our results show a transition in myosin VI stepping behaviour in response to ADP and load. Further, the results indicated two binding states: the distant binding state, where the two heads span the actin helical pitch (36 nm) when bound to the actin filament (typically observed in hand-over-hand steps), and the adjacent binding state, where the two head are close (<10 nm apart) when bound to the filament (typically observed in inchworm-like steps). These two states are consistent with previous reports^23^,^34^. In the stalled state, we observed that myosin VI repeatedly transitioned between the distant binding and adjacent binding states (Fig. 4b, dashed-line box). The probabilities of short forward and backward steps were almost equal (Fig. 3i), similar to the stalled state of the Brownian ratchet, showing myosin VI is not completely locked onto the actin filament at the stalled state. This dynamic binding and release of the head cannot be observed in conventional force measurement systems such as optical tweezers, as these assays monitor the tail position^20^,^22^.

The adjacent binding state contributed more to the stalled state than did the distant binding state (Fig. 4c). This is in contrast to the bound state in the absence of load, where the distant binding state is dominant (Fig. 4d). The adjacent binding state may evenly distribute the external load to both heads, which could reduce the risk of diffusing away from the actin. Supporting this notion, we found only 26% of detachments from actin occurred in the adjacent binding state (12 observations/46 detachment events). Consequently, efficient anchoring of myosin VI can be promoted by both the load dependence of the ATP hydrolysis cycle^20^,^22^ and by the load dependence of the bias towards the adjacent binding state.

## Discussion

The combination of our DNA nanospring technology with SMF imaging reveals that higher forces induce a transition from hand-over-hand to inchworm-like motion in myosin VI. Furthermore, the adjacent binding state was observed to be predominant at stall force, which is consistent with myosin VI anchoring in the cell. These two stepping patterns (or bound states) and the transition between them can only be distinguished by independent tracking of the two head domains under load. The results demonstrate that our DNA nanospring system can reveal the force-dependent functional changes of molecular machines.

**Figure 5:**
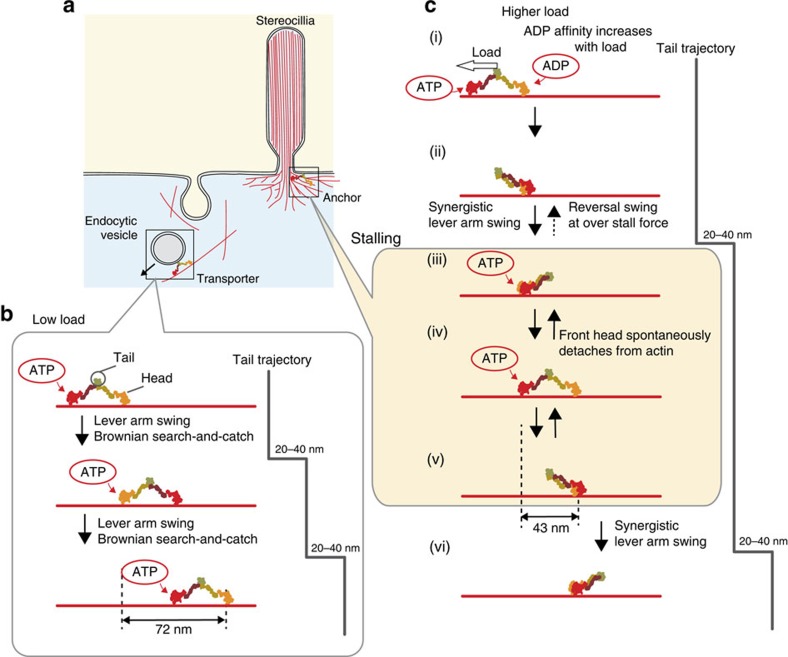
Force-dependent transport and anchoring mechanisms of myosin VI. (**a**) Examples of myosin VI transport and anchor function in cells. Endocytic vesicle transport and the maintenance of stereocillia are shown as examples. (**b**) Hand-over-hand motion is used for transport, which occurs under low loads. Here, one head in a myosin VI dimer undergoes one 72 nm step per two successive ATP hydrolysis cycles^30^, whereas the tail moves 20–40 nm per ATP hydrolysis cycle^32^. (**c**) Inchworm-like motion occurs at high loads. Here, one head undergoes 43 nm steps per one ATP hydrolysis cycle (states i and ii). In the adjacent binding state (state iii), either head can take the forward step with equal probability^34^, which results in a synergistic lever arm swing (state v to vi) to produce 20–40 nm tail motion. At stall force, myosin VI remains at state iii, iv or v. Transitions from state iv to iii or v to iv should result from spontaneous detachment of the front head^49^. Transitions from state iii to ii would be possible at over stall force (>2.5 pN), resulting in backward motion by the tail^22^.

We summarize the stepping mechanism of myosin VI under varying loads in Fig. 5. At low load, the hand-over-hand mechanism, where non-adjacent binding states are occupied, is used for the efficient transport of cargos in the cell, because it converts the chemical energy of one ATP into a large forward step (∼72 nm head trajectory or 20–40 nm tail trajectory/ATP molecule hydrolysed^32^) (Fig. 5b). In contrast, an inchworm-like mechanism, where adjacent binding states are occupied half the time, would be inefficient for transport at low load, because it consumes two ATP molecules to produce two short forward steps (43 nm head trajectory each step) to obtain a 20–40 nm tail trajectory^23^,^34^ (Fig. 5c). At higher loads, the stepping mechanism transitions to inchworm-like steps, which may be more efficient for transport, because Brownian ratchets can oppose larger forces when steps are smaller. At the stall force, the myosin heads continue to bind and release, visiting both adjacent and distant binding states (Fig. 4a,b). This is unexpected in terms of the physiological role as an anchor, because robust binding to actin is ideal for anchoring and, in fact, some invertebrate smooth muscles robustly catch actin over long periods with little energy expenditure^36^. We speculate the dynamic binding and release observed in myosin VI at stall force is useful as an adaptation to changes in membrane tension. This feature is similar to skeletal muscle, where dynamic binding and release is thought to allow for quick rearrangement of the binding sites in response to a sudden change in length, reducing or recovering tension^37^.

How does the observed transition to inchworm-like stepping occur at physiological ADP and loading condition? We know that ADP affinity of the heads increases with load^20^, and that when in the ADP bound state, the front head prefers the non-tilted-forward lever arm state^25^ (state ii in Fig. 5c and reported by Menetrey *et al*.^38^). Thus, under load, the front head is more likely to be in the ADP bound state, which is less likely to tilt forward, and it has been suggested that this leads to more frequent inchworm-like steps^23^. This mechanism is consistent with our observation of a transition to inchworm-like stepping under load with physiological ADP.

The nanospring is a bottom-up force-application nanoscale device, which does not require large-scale equipment, unlike conventional force-application biophysical tools, such as OT, AFM and fluidic chambers. Because of this, our system provides a simple and flexible approach to study single-molecule events under variable force. Our assay system can be extended to other actin-based and microtubule-based motor proteins (myosin II, kinesin and dynein and so on) so long as the ssDNA is labelled to the target proteins. For microtubule-based motors, mutant kinesin^39^ may be used as the anchor for the microtubule. Furthermore, our technique is compatible with other single molecule observation systems (for example, high-speed AFM, dark-field microscopy and electron microscopy), which significantly expands the range of potential biophysical experiments. For example, high-speed AFM observation^25^ of macromolecules tethered with a nanospring could directly elucidate the force dependence of their structural dynamics and stability. Similarly, dark-field imaging of GNPs tagged with a macromolecule^23^,^40^ and the nanospring would enable us to observe the subdomain motion of proteins perturbed by force with high spatio-temporal resolution. In addition, our nanospring is highly compatible with other DNA origami nanostructures, such as synthetic cargo (or ‘chassis')^41^, which can be used to control motor protein assembly, and could be used to investigate the load-dependent mechanical properties of such assemblies. More generally, our DNA nanospring has a tunable spring constant and can be attached to various types of molecules, allowing for the control of species, number, spacing and orientation during force-dependent measurements (for example, Supplementary Fig. 7). The programmability of nanospring–motor protein complexes could also be the basis for nanoactuators to study other mechanosensory proteins or be used as components for synthetic nanoscale devices that require dynamic buildup of mechanical potential energy.

## Methods

### DNA origami spring (nanospring)

Nanosprings were designed using caDNAno software (Supplementary Fig. 1, http://cadnano.org and ref. 42) and three-dimensional structures were produced by CanDo, which is a free online resource to predict the 3D solution shape of DNA origami (Fig. 1a, http://cando-dna-origami.org/ and ref. 43).

To fold the spring, 10 nM of scaffold was mixed with 100 nM of core staples (Supplementary Data). Oligos were obtained from Hokkaido System Science or IDT. The folding reaction was carried out in Folding buffer (5 mM Tris pH 8.0, 1 mM EDTA and 2–30 mM MgCl_2_) with rapid heating to 80 °C and cooling in single degree increments to 60 °C over 2 h followed by additional cooling in single degree increments to 25 °C over another 2 h.

The folded nanospring was purified by agarose gel electrophoresis. Structures were loaded into 2% agarose gels and run at 50 V for 3 h in TBE buffer (45 mM Tris, 45 mM boric acid and 1 mM EDTA) supplemented with 11 mM MgCl_2_. The highest migration bands (see Supplementary Fig. 2) were excised, crushed and spun through a Freeze and Squeeze column (Bio-Rad) for 10 min at 20,000 *g* and 4 °C. The nanospring was stored at 4 °C.

The purified nanospring was adsorbed for 5 min onto glow discharged formvar- and carbon-coated copper grids, stained for 1 min with 2% uranyl formate and 25 mM NaOH. Images were acquired at 80 kV under low-dose conditions in a Tecnai T12 equipped with a LaB_6_ filament and a 4k × 4k CCD (charge-coupled device) camera. The nominal magnification was × 50,000 for a pixel size of 3.66 Å at the sample level.

### Bead-nanospring conjugation

To estimate the force-extension curve of the nanospring, biotin-modified staples and digoxigenin (DIG)-modified staples (Supplementary Data) were added to the 100 nM core staples, which were then folded to attach biotin and DIG at opposite ends of the nanospring.

Carboxylate-modified polystyrene beads (0.2 μm in diameter, Invitrogen) were crosslinked to anti-DIG polyclonal antibody (Roche) and BSA using the Polylink Protein Coupling Kit (Polysciences, Inc.). One microlitre of anti-DIG antibody-coated beads (∼3 nM) and 10 μl of nanospring (∼1 nM) were mixed and incubated for 1 h on ice before use in an optical tweezers assay.

A single flow chamber was made using double-sided transparent tape (Scotch) and coverslips (Matsunami). Five microlitres of neutravidin (5 mg ml^−1^, Invitrogen) was flowed into a chamber and incubated for 3 min. Unbound neutravidin was washed out by Assay buffer (AB; 20 mM HEPES-KOH pH 7.8, 25 mM KCl, 5 mM MgCl_2_ and 1 mM EGTA) and 10 μl of biotin-coated bead (∼3 pM and 0.2 μm in diameter, Invitrogen) was flowed into the cell, which was then incubated for 5 min. Twenty microlitres of bead-nanospring (bead-NS) was diluted ten times in AB plus an oxygen scavenger system^44^ and flowed into the cell, which was then sealed with nail polish. The chamber was observed by our microscope after 5 min.

### Optical tweezers assay

Fluorescence images of bead-NS were detected by an electron modifying CCD (EMCCD) camera (DL658M-NIT or DU860D-CS0, Andor) at a 30 Hz or 300 Hz recording rate. Bead-NS underwent tethered Brownian motion and an isotropically diffusing bead-NS was chosen (see Supplementary Fig. 3c) for analysis, to avoid the effects of nonspecific interactions with the glass surface. The bead-NS motion was recorded for 500 frames by the EMCCD camera and the fluorescence images were fitted by a 2D Gaussian distribution to precisely determine the centre position of the fluorescent bead with nanometre accuracy by FIONA^30^ and a laboratory-written Labview programme (National Instruments). We estimated the fix point of bead-NS on the glass surface as the mean value of the probability density distribution of the bead position.

When the OT centre was positioned in the vicinity of the fix point, the trapping laser (J15-BL-RW, Spectra-Physics Lasers, Inc.) was switched on to capture the bead (Supplementary Fig. 3d, left). A piezo-driven stage (p-733.3DD, Physik Instrumente) was moved by a triangle wave (frequency, 0.2 Hz or 10 Hz; amplitude, 2.5 μm) in one axis and the trapped bead image was tracked by FIONA. We obtained the force-extension curve according to the procedure described in Supplementary Fig. 3.

### Design of myosin constructs

Human myosin Va complementary DNA (Kazusa Product ID ORK07567) was truncated at Gly924. This fragment included the motor domain, lever arm domain (IQ1-6) and a small part of the coiled-coil domain (11 a.a., SVERYKKLHIG). HaloTag (Promega) was attached at the N-terminal via a linker (15 a.a., LRRRPTRPAMDPPSK). For oligo labelling and protein purification, SNAP-tag (NEB) and 6 × His-tag were attached at the C-terminal via linkers (2 a.a., RA). This Halo-hMyosinVa-SNAP-His fragment and the human calmodulin gene (Met1-Lys149, Kazusa Product ID ORK01403) were introduced downstream of the PH and p10 promoters of the Baculovirus expression vector pFastBac Dual, respectively.

Human myosin VI cDNA (Kazusa Product ID ORK01080) was truncated at Ala1021. This fragment included the motor domain, calmodulin-binding domain, and the proximal, medial and most of the distal tail domain (41 a.a., IQAEVEAQLARQKEEESQQQAVLEQER RDRELALR IAQSEA). For QD labelling, HaloTag was attached at the N-terminal via a linker (15 a.a., LRRRPTRPAMDPPSK). For oligo labelling and protein purification, SNAP-tag (NEB) and 6 × His-tag were attached at the C-terminal via linkers (2 a.a., RA). This Halo-hMyosinVI-SNAP-His fragment and the human calmodulin gene (Met1-Lys149, Kazusa Product ID ORK01403) were introduced downstream of the PH and p10 promoters of the Baculovirus expression vector pFastBac Dual, respectively.

### Protein expression and purification with oligonucleotide labeling

Monomeric forms of myosin V and VI were expressed and purified as follows. Recombinant viruses for myosin V or VI heavy chain and calmodulin were produced by homologous recombination using the Bac-to-Bac Baculovirus Expression System (Life Technologies). Sf9 insect cells were maintained in a monolayer culture in ventilated 175 cm^2^ flasks at 28 °C with 10% fetal bovine serum and 1 × Antibiotic-Antimycotic liquid/Sf-900 II SFM (Life Technologies). After 60 h of incubation for recombinant proteins expression, cells were collected by centrifugation at 6,000 *g* for 5 min and stored at −80 °C. Frozen cells were suspended and sonicated in 2 ml Lysis buffer (30 mM Tris–HCl pH 8.0, 200 mM NaCl, 1 mM EGTA, 5 mM MgCl_2_, 5 mM ATP and 10 mM 2-mercaptoethanol) containing Complete, EDTA-free protease inhibitors cocktail tablet (Roche Diagnostics) per flask of cells. After ultracentrifugation at 35,000 *g* for 20 min, soluble fractions were mixed with pre-washed 100 μl Ni-NTA Agarose (QIAGEN) for 40 min. Afterwards, wash buffer (20 mM Tris–HCl pH 8.0, 300 mM NaCl, 0.2 mM EGTA, 1 mM MgCl_2_, 1 mM ATP, 10 mM 2-mercaptoethanol and 20 mM imidazole pH 8.0) was added into the column, to remove unbound proteins. Myosin was eluted by Elution buffer (20 mM Tris–HCl pH 8.0, 100 mM NaCl, 0.2 mM EGTA, 1 mM MgCl_2_, 10 mM 2-mercaptoethanol and 300 mM imidazole pH 8.0) and myosin V or VI recombinants were obtained (0.3–1 mg ml^−1^).

Oligo labelling reactions were performed just after His-tag affinity purification. Amine-modified DNA oligos (Hokkaido System Science) (Oligo A*, B*, C* and D* in Supplementary Data) were linked to the SNAP substrate, benzylguanine (BG; NEB), and 25 μM of BG-oligonuculeotides was labelled with ∼1 μM myosin V or VI containing an C-terminal SNAP_f_ tag (NEB) in His tag affinity Elution buffer for 30 min at room temperature. Oligo-labelled myosin V and VI were purified by actin filament affinity, aliquoted and stored at −80 °C until use. Just before the experiment, ∼1 μM oligo-labelled monomeric myosin V or VI was mixed and incubated for 30 min at 4 °C, to form DNA-hybridized myosin dimers. The efficiencies of labelling the oligos to and the dimerization of myosin VI were estimated by a gel-shift assay (4–15% gradient gel, Biorad) and determined as >90 and >30%, respectively.

### Immobilization of myosin II

Myosin II was extracted and purified from rabbit skeletal muscle^44^. Heavy meromyosin (HMM) was obtained by enzymatically digesting myosin with α-chymotrypsin^45^. Amine-modified DNA oligo (100 μM; Hokkaido System Science; Oligo D* in Supplementary Data) was mixed with 50 mM sulfo-SMCC (Thermo Scientific) and incubated for 1 h at room temperature. Excess SMCC was removed three times by gel filtration (Micro BIO-SPIN P-6, Biorad). HMM was mixed with SMCC-oligo at the stoichiometry 1:10, incubated overnight at 4 °C and then mixed with *N*-ethylmaleimide at 1:2 for 30 min at 4 °C. Excess SMCC-oligo was removed twice by ultrafiltration (Amicon Ultra 50 K, Merck Millipore) and Oligo D*-labelled HMM was aliquoted and stored at −80 °C until use. Labelling efficiency was estimated to be ∼50% by a gel-shift assay. Immobilization was confirmed by the *in vitro* motility assay^46^.

### Myosin-QD conjugation

Qdot 565 amine-derivatized polyethylene glycol (PEG) conjugates (4 μM; Life Technologies) was mixed with 50 mM HaloTag succinimidyl Ester (O4) ligand (Promega) and incubated for 1 h at room temperature. Excess HaloTag ligand was removed three times by gel filtration (Micro BIO-SPIN P-6, Biorad) and the HaloTag ligand-QD conjugation was stored at 4 °C.

Myosin VI dimer (2 μl; ∼500 nM) was mixed with 1 μl HaloTag ligand-QD conjugation (∼2 μM) and incubated for 3 h on ice before use for single-molecule imaging experiments. In the case of dual colour imaging, myosin VI monomer was used when mixing with QD. Then, just before the experiment, ∼500 nM oligo-labelled monomeric myosin VI with Qdot 565 or 655 was mixed together and incubated for 30 min at 4 °C to form DNA-hybridized myosin VI dimers labelled with different colour QDs.

### Myosin-NS conjugation

For the myosin-NS conjugation, handle staples (Supplementary Data) were added to the 100 nM core staples, which were then folded to attach optional 21 bp ssDNA to both ends of the nanospring. One microlitre of DNA-hybridized myosin dimer or immobile myosin II (0.5∼1 μM) with anti-handle oligos was mixed with 10 μl of nanospring (∼10 nM condensed by ultrafiltration (Amicon Ultra 100 K, Merck Millipore)) and incubated for 30 min on ice.

### Single-molecule imaging

To avoid nonspecific interactions of myosin-NS with the glass surface, a glass coverslip was coated with functional PEG according to Schroeder *et al*.^47^ with some modifications. Briefly, coverslips were cleaned with a plasma cleaner for 10 min (FEMTO, Diener electronic). The coverslips were then soaked in freshly prepared 2% (3-Aminopropyl) trimethoxysilane (KBM-603, Shin-etsu Chemical) in acetone for 45 min with gentle shaking at room temperature and rinsed with ddH_2_O. Coverslips were dried and 30 μl of PEG solution (100 mg ml^−1^ (PEG 5000 (ME-0500HS, NOF Corp.):PEG 3400 (DE-034HS, NOF Corp.):biotin-PEG (BI-050TS, NOF Corp.)=100:10:1) in freshly prepared 0.1 M bicarbonate buffer pH 8.3) was put between a pair of coverslips and incubated for 3 h. After rinsing with ddH_2_O, 20 μl of sulfodisuccinimidylartrate (Solteck Ventures) solution (30 mg ml^−1^ in freshly prepared 1 M bicarbonate buffer pH 8.3) was put between a pair of coverslips and incubated for 45 min. The coverslips were rinsed, dried and stored at −80 °C in a vacuum desiccator with desiccant.

A single flow chamber was made using double-sided transparent tape (Scotch) and PEG-coated coverslips. Five microlitres of neutravidin (Invitrogen, 0.25 mg ml^−1^) was flowed into a chamber and incubated for 3 min. Unbound neutravidin was washed out by AB. Ten microlitres of biotinylated actin filament solution (0.01 mg ml^−1^, 10% biotinylation) was flowed into the cell and incubated for 5 min. Unbound actin was washed out by AB and 10 μl of Blocking buffer (10 mg ml^−1^ BSA, 0.1% Tween 20 in 5 mM EDTA, 10% glycerol, 0.3 M NaCl and 0.1 M phosphate buffer pH 7.5) was flowed into the chamber, which was then incubated for 2 min. The chamber was then washed with 30 μl of Motility buffer (AB plus 2 mM ATP (Oriental Yeast); 0.436% ADP contaminants ∼8 μM in 2 mM ATP) and an oxygen scavenger system^44^. For 0 μM ADP experiments, stocked ATP was purified by liquid chromatography^22^. An ATP regeneration system^48^ was added in the 0 μM ADP experiments and 100 μM ADP was added in the 100 μM ADP experiments. Ten microlitres of myosin-NS or myosin-QDs-NS diluted 10 or 100 times in Motility buffer was flowed into the cell. The chamber was sealed with nail polish and observed immediately.

Single-molecule imaging and analysis of the nanosprings or QDs was performed using TIRFM. Illumination was provided by 488 nm laser light (OBIS 488LS-100, Coherent), 532 nm laser light (Compass 315M-100, Coherent) or 640 nm laser light (OBIS 640LX-75, Coherent). The fluorescence of TAMRA labelled with nanospring or Qdot 565 labelled with myosin was passed through a dichroic mirror (FF552-Di02 or FF506-Di03, Semrock) and emission filter (FF01-585/29 or FF01-562/40, Semrock). In the case of dual colour imaging, the fluorescence of Cy3 and ATTO647N labelled with nanospring or Qdot 565/655 labelled with myosin was passed through a dichroic mirror (FF01-577/690-25 or FF552-Di02) and a dual-view apparatus (Hamamatsu) equipped with dichroic mirrors (DML630 nm or DML 600 nm, Asahi Spectra), and emission filters (FF01-585/29 or FF01-562/40 and FF655/15, Semrock) were put in front of the EMCCD camera (Andor, DV887ECS-BV). TAMRA-labelled nanosprings and QDs showing smooth processive motions were chosen for the analysis and a sufficient data set was collected from multiple preparations (flow chambers) to estimate the fit values.

Accurate and precise co-localization of different coloured fluorescent probes was achieved in accordance with SHREC^35^. Briefly, multicolour fluorescent beads (Ultra Rainbow Fluorescent particles, 0.2 μm, Spherotech) stuck on a coverslip were used as fiducial markers for each imaging channel. An alignment grid by the fluorescent beads was created with a piezo stage (Physik Instrumente, P-517. 3CD) and the fiducial registration error for each marker was calculated by MATLAB software and estimated to be <7 nm over 12 h, which ensured sufficient stability during the single-molecule experiments.

### Photobleaching assay

To quantify the number of myosin VI attached to a nanospring, 10 μM HaloTag TMR ligand (Promega) was labelled with ∼1 μM myosin VI during the labelling process of 21-mer BG-oligo. Labelling efficiency was spectrophotometrically estimated to be >95%. The TMR and 21-mer oligo-labelled myosin VI was mixed with 42-mer oligo-labelled myosin VI and incubated for 30 min at 4 °C to form myosin dimers. Myosin-NS was prepared as described in Myosin-nanospring (myosin-NS) conjugation section and purified with Sephacryl S-500 HR resin (GE Healthcare) twice. The resulting myosin-NS was diluted in Motility buffer, flowed into the cell and observed by TIRFM.

### Analysis

The force–velocity relation and the precision of the nanospring extension were determined using Origin 8.5 (Origin Lab.). For the force–velocity relation, individual displacements obtained from tracking a fluorescently labelled nanospirng were averaged to reduce Brownian noise. Line fits were made to successive 1 s time segments and the velocity for each segment was determined as well as the mean force during the segment. Data points were clustered at fixed intervals of force and the average velocities were plotted against average force. The s.e. of the force includes the error from the force-extension curve (average standard deviation is 0.19 pN at 0–2 pN range). The force–velocity curve was constructed from 38 processive motions.

To estimate the precision of the nanospring extension, traces obtained from a single 2D Gaussian fit of TAMRA nanospring images at low ATP concentration (20 μM) were analysed. The precision was defined as the s.d. of the centre positions in a 1 s window. Traces at low load regimes (<500 nm extension) were excluded from the analysis, because the myosin VI stepping could have affected the precision (the stepping rate was 1.1 s^−1^ at 20 μM ATP and without load^23^). We plotted the precision versus the nanospring extension (Supplementary Fig. 4).

### Data availability

The authors declare that the data supporting the findings of this study are available within the article and its Supplementary Information files, or are available from the authors upon request.

## Additional information

**How to cite this article:** Iwaki, M. *et al*. A programmable DNA origami nanospring that reveals force-induced adjacent binding of myosin VI heads. *Nat. Commun.* **7,** 13715 doi: 10.1038/ncomms13715 (2016).

**Publisher's note**: Springer Nature remains neutral with regard to jurisdictional claims in published maps and institutional affiliations.

## Supplementary Material

Supplementary Information Supplementary Figures 1-7 and Supplementary References

Supplementary Data Scaffold, Core staples, Handles and Anti-handles for nanospring

Supplementary Movie 1 A 200 nm fluorescent bead tagged with a nanospring (right bead) was optically trapped at the fixed trap center, and the cover glass was moved by a piezo-driven stage (triangle wave, 0.2 Hz frequency, 2.5 μm amplitude). The displacement of the cover glass was calculated using a fluorescent bead stuck on the cover glass (left bead). The fluorescence image was recorded by an EMCCD camera at a 30 Hz recording rate. See also Supplementary Fig. 3.

Supplementary Movie 2 A nanospring was fluorescently labeled by thirteen TAMRA-labeled staple strands, and immobile myosin II and single myosin VI were attached to the ends. Non-fluorescent actin filaments were adsorbed on the cover glass, and the nanospring-motor protein complex was tethered to the actin via immobile myosin IIs. Myosin VI tethered with the nanospring moved processively along actin filaments, and the stretch/compression dynamics of a fluorescently labeled nanospring could be seen by TIRF microscopy (10 Hz frame rate, ×10 play). See also Fig. 2d.

Supplementary Movie 3 Both ends of a nanospring were fluorescently labeled and attached to single myosin V and VI molecules (Cy3 for myosin VI, indicated by dark green; ATTO647N for myosin V, indicated by red). Dual-color imaging was done by TIRF microscopy, and the two imaging channels were co-localized by SHREC (10 Hz frame rate, ×1 play). See also Fig. 3a.

Supplementary Movie 4 A myosin VI monomer was labeled with Qdot (Q565) via HaloTag at the N-terminal head domain and dimerized with a non-labeled myosin VI monomer via DNA hybridization. The resulting dimer moved processively along actin filaments, and the fluorescence image was observed using TIRF microscopy (30 Hz frame rate, ×20 play). See also Supplementary Fig. 5.

Supplementary Movie 5 Qdot (Q655)-labeled myosin VI (indicated by red) was tethered to an actin filament via a TAMRA-labeled nanospring (indicated by dark yellow), and the fluorescence image was observed by TIRF microscopy (10 Hz frame rate, ×5 play). Myosin VI exhibited processive motion, stalling and detachment from actin. See also Fig. 3c.
