# Supplementary material for: A programmable DNA origami nanospring that reveals force-induced adjacent binding of myosin VI heads
Source: Nat Commun. 2016 Dec 12;7:13715. doi: 10.1038/ncomms13715 (PMC5159853; doi:10.1038/ncomms13715)
Supplement: Supplementary Information — Supplementary Figures 1-7 and Supplementary References [file ncomms13715-s1.pdf]

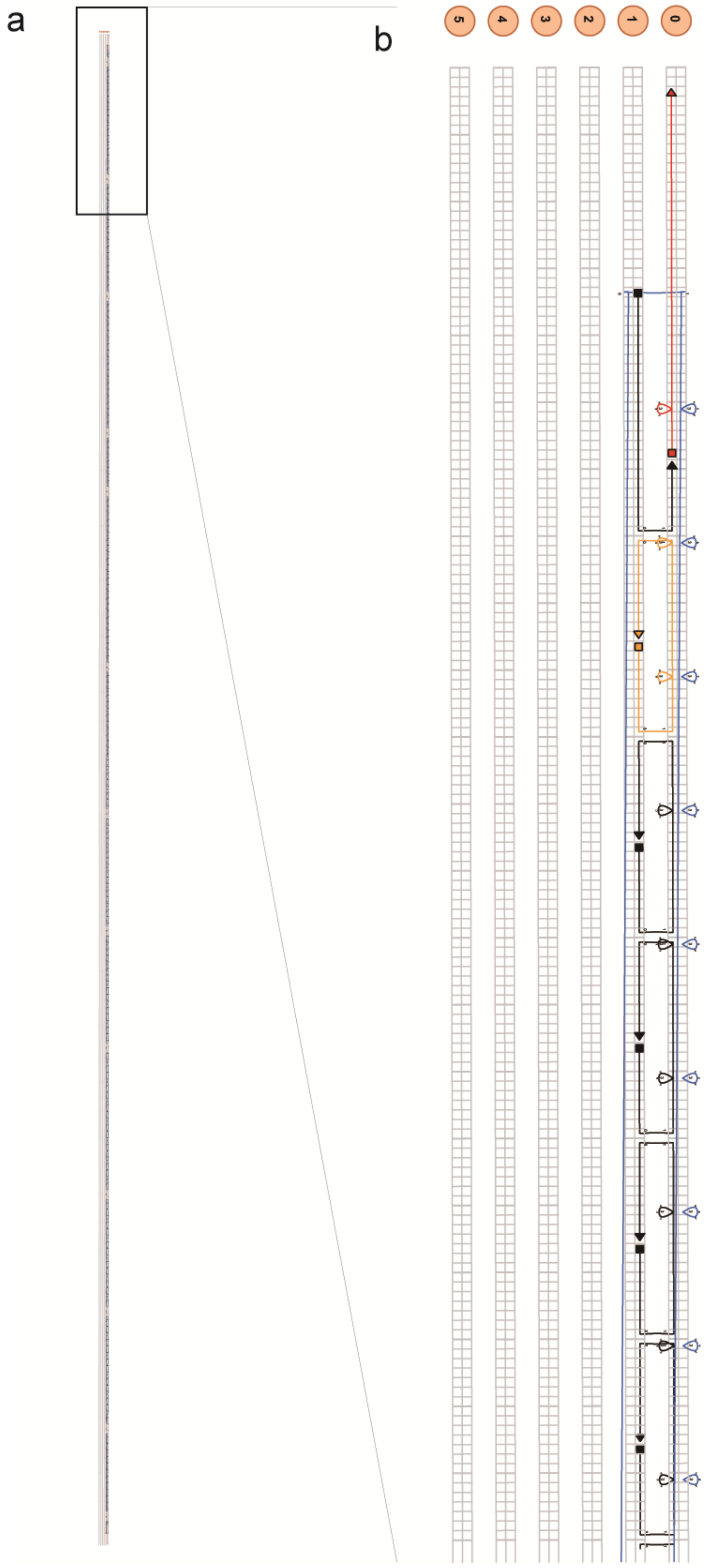
a
b
0
1
2
3
4
5

Supplementary Figure 1

Design of the DNA origami spring (nanospring).

(a) Overview of the 2-helix bundle (2HB) nanospring design used in this study. The scheme was produced by caDNAno software[1]. Scaffold, core staples, handles for linking with myosin and TAMRA-labeled staples are shown in blue, black, red and orange, respectively. The boxed area is expanded in (**b)**. Normally, two double helices (indicated by "0" and "1" in **(b)**) were bundled via crossover staples per 21 bp to avoid internal strain. However, to produce negative superhelical strain[2] to form a coil structure, 3 base pairs (bp) were additionally inserted every 14 bp in helix "0" (indicated by loops).

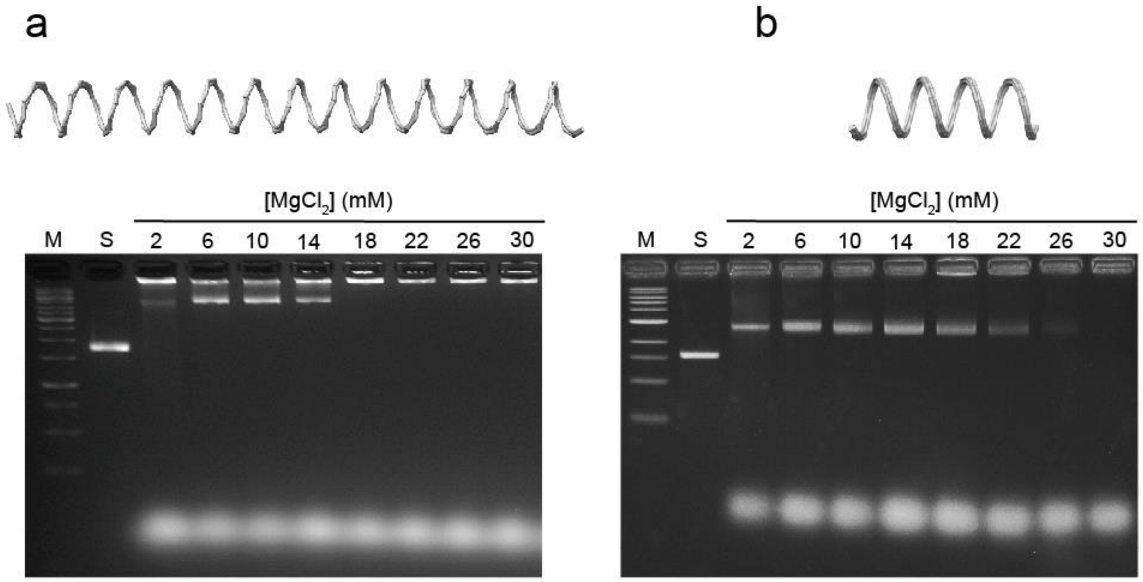

Supplementary Figure 2

Gel purification of 2 HB and 4 HB nanosprings.

(a) Test for folding of a 2HB nanospring at different Mg^{2+} concentrations (2-30 mM) by agarose gel electrophoresis (AGE). Increasing agarose-gel mobility correlated with improved quality of folding, suggesting that correctly folded structures tend to be more compact than misfolded versions[3]. Therefore, the fastest migration band in the 14 mM $MgCl_2$ lanes was purified and used for transmission electron microscopy imaging and single molecule experiments. M: 0.5-10.0 kb DNA ladder (New England Biolabs); S: Scaffold in 5 mM Tris, 1 mM EDTA and 11 mM $MgCl_2$. (**b**) Test for folding of a 4HB nanospring.

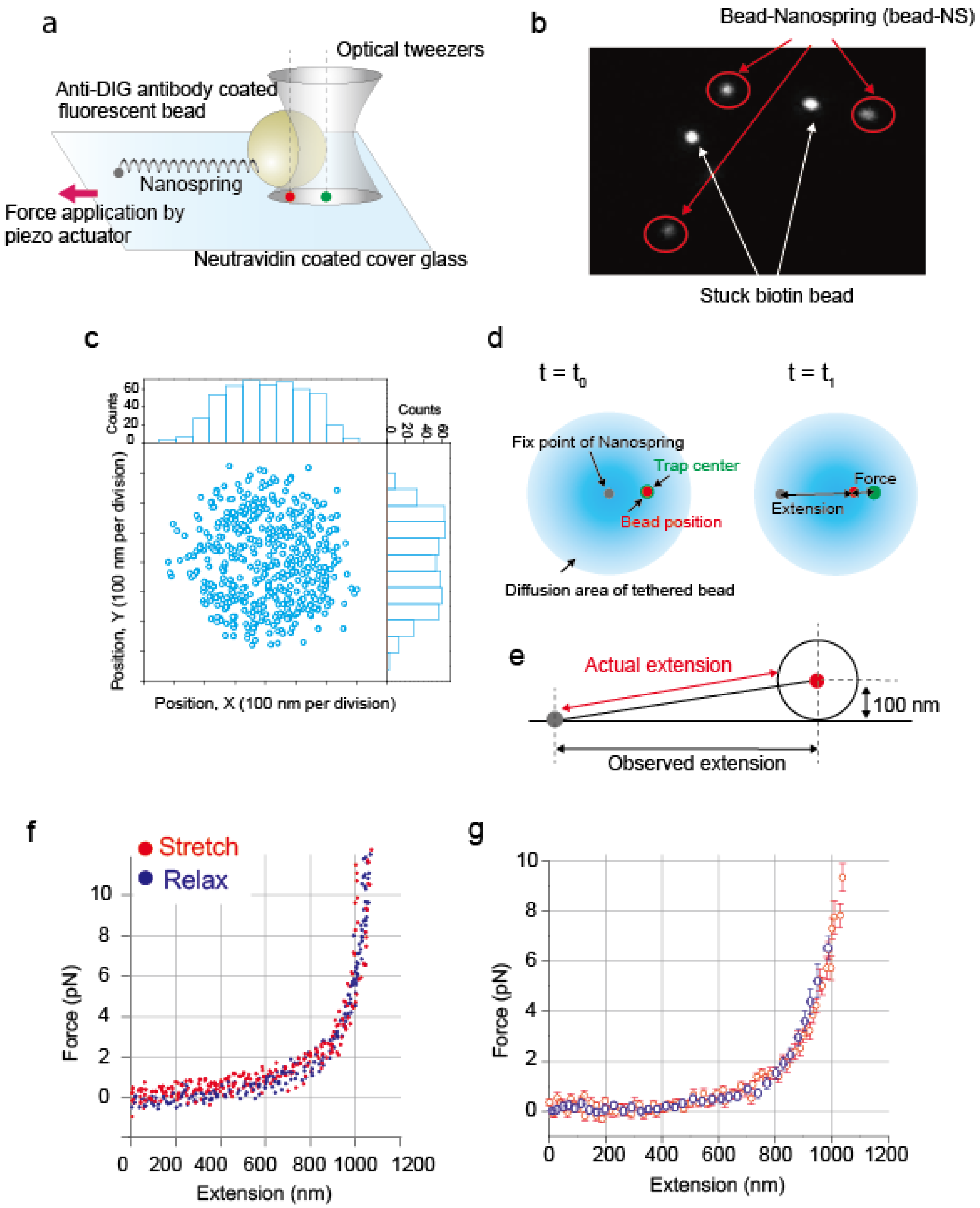

Supplementary Figure 3

Force-extension curve of nanosprings.

(a) Experimental setup for the optical tweezers assay. A 200 nm fluorescent bead tagged with a nanospring (bead-NS) was optically captured, and the cover glass was then moved

by a piezo actuator. Grey circle, fix point of the nanospring on the cover glass set by a biotin-neutravidin bond; red circle, center position of the fluorescent bead; green circle, center position of the optical trap. (**b)** Fluorescence image of bead-NS attached to the glass slide. Unlike a stuck biotin coated bead, limited diffusion can be seen by bead-NS (red circles). (**c)** 2D mapping of the bead-NS position by FIONA. The fix point of the nanospring was determined from the probability density. (**d)** Left, a bead-NS free to diffuse in two dimensions was optically captured within a few pixels of the fix point (< 100 nm). Right, the piezo stage was moved, and the extension of the nanospring and force applied were estimated. To estimate the extension, the fix point (grey circle) was calculated by tracking a stuck biotin coated bead to precisely determine the displacement of the cover glass. To estimate the force, we calculated the distance between the bead position (red circle) and trap center (green circle) and multiplied by the trap stiffness. (**e)** The observed extension was corrected by considering the bead size and 3D geometry as follows.

$$E_{extension} = \sqrt{{E_{observed}}^2 + 100^2} - 100$$

(f) A force-extension curve of the 2HB nanospring measured by optical tweezers shows reversible stretches (red circles) and relaxation (blue circles). **(g)** Comparison of the force-extension curve at different stretching rates. Red circles are the same as Figure 1d (stretching rate, 1 nm/ms) and blue circles were obtained at the stretching rate 50 nm/ms (N=10).

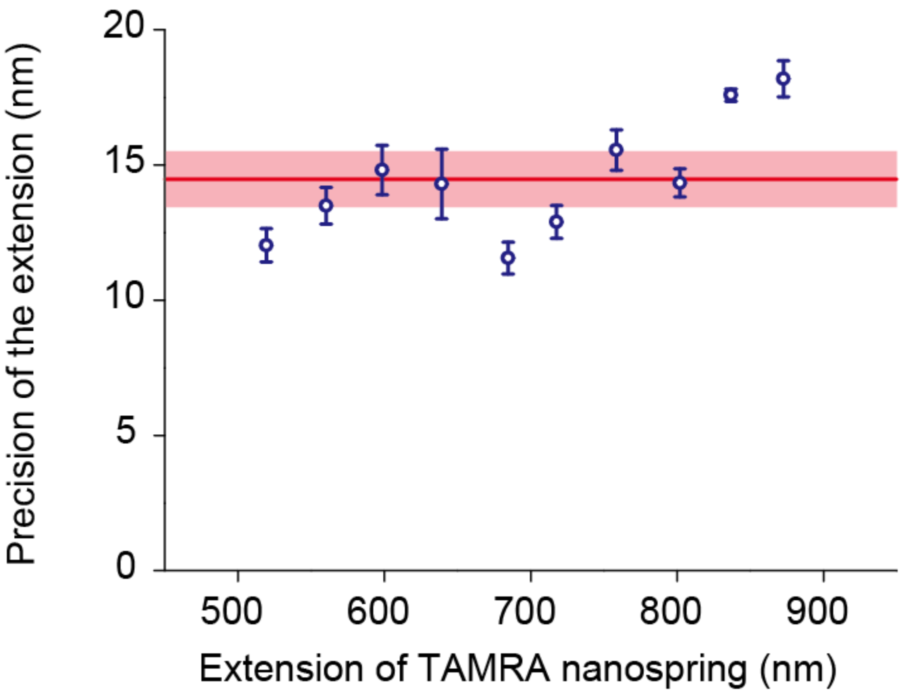

Supplementary Figure 4

Precision of the nanospring extension versus extension.

The precisions of the extensions of 11 nanospring (standard deviation of the mean position of nanospring multiplied by two) were plotted against the nanospring extension. Blue points with error bars indicate mean ±s.e.m. The weighted mean value, 14 nm, is shown as a horizontal red line with standard deviation (2.0 nm) shown in light red. The number of data points for each plots is N=88-273.

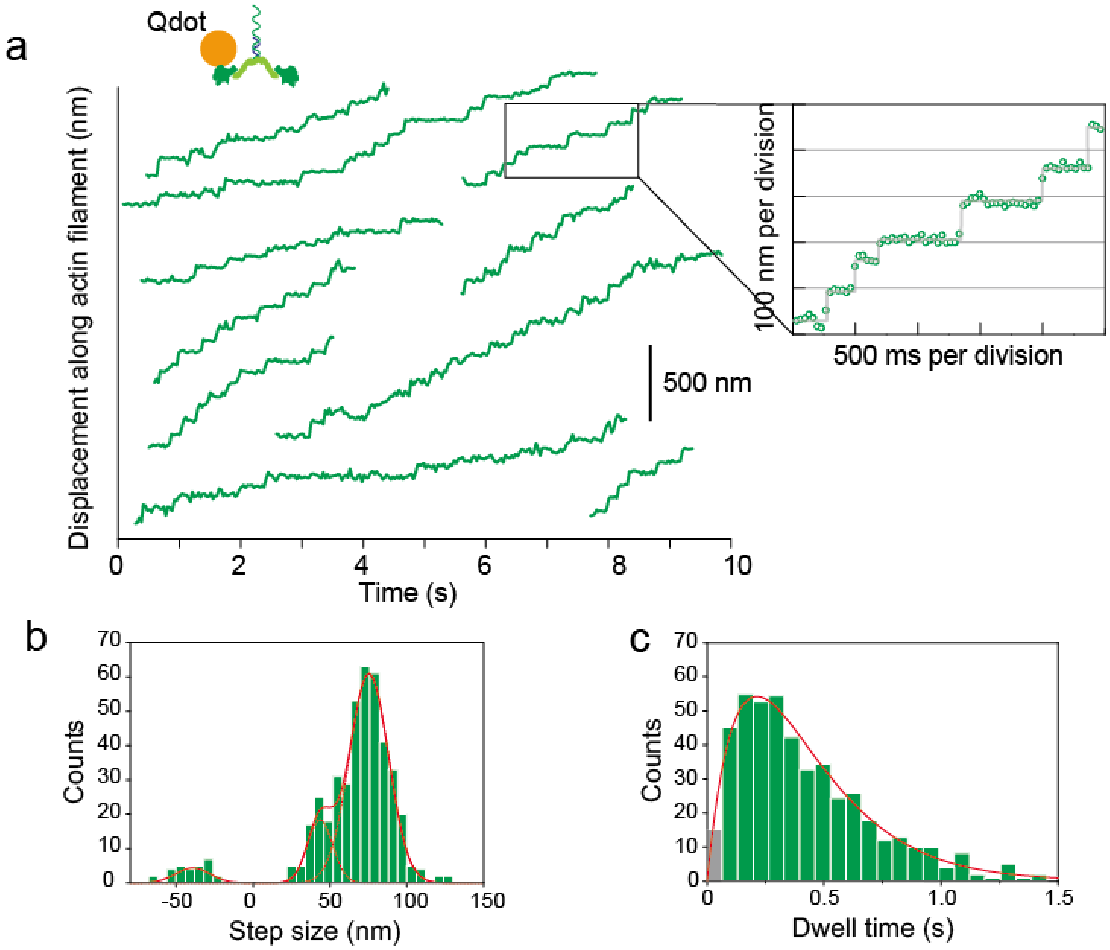

Supplementary Figure 5

Stepping dynamics of DNA hybridized myosin VI dimer.

(a) Stepping trajectories in the absence of a nanospring at 2 mM ATP. Inset is an expanded view of the boxed area. The step sizes and dwell times of adjacent steps are shown in **(b)** and **(c)**, respectively. The red Gaussian fit in **(b)** represents three steps (-39.9 ± 11, 43.5 ± 8.1, 75.3 ± 12 nm; mean ± S.D.); the grey bar (0-66 ms) in **(c)** indicates reference data due to the limited time resolution (33 ms).

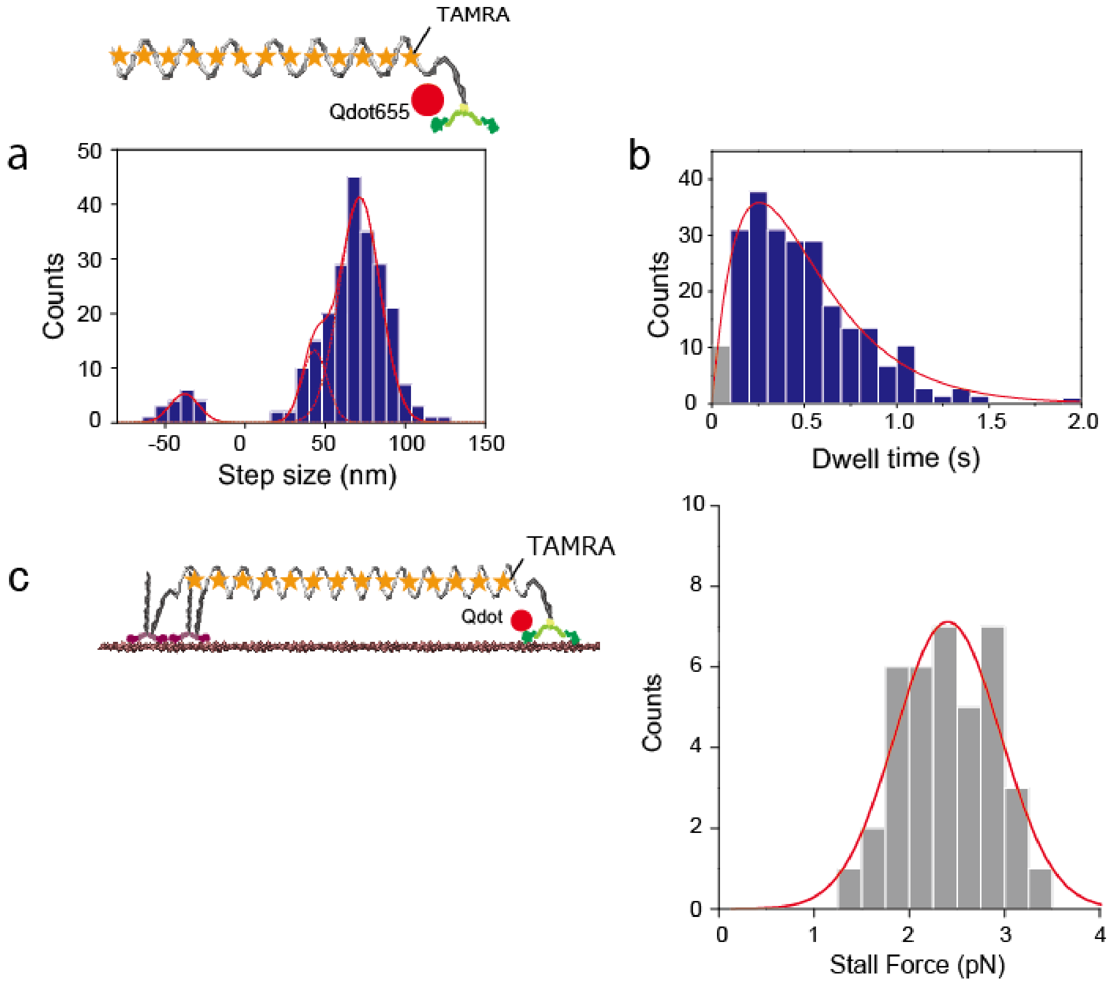

Supplementary Figure 6

Stepping dynamics and stall force of Qdot-labeled myosin VI dimers with nanosprings.

(a) and **(b)** The step sizes and dwell times of single myosin VI dimers tagged with TAMRA-labeled nanosprings. The red Gaussian fit in **(d)** represents three steps (-37.9 ± 9.1, 43.2 ± 7.8, 71.5 ± 12 nm). The dwell time distribution in **(b)** was fit to the formula $tk^2exp(-kt)$, with $k = 3.9\ s^{-1}$. **(c)** Stall forces of single Qdot-labeled myosin VI tagged with a nanospring. The distribution fit to the Gaussian function is shown (red line, 2.4±0.5 pN (mean±S.D.)). The forces were estimated from Qdot trajectories.

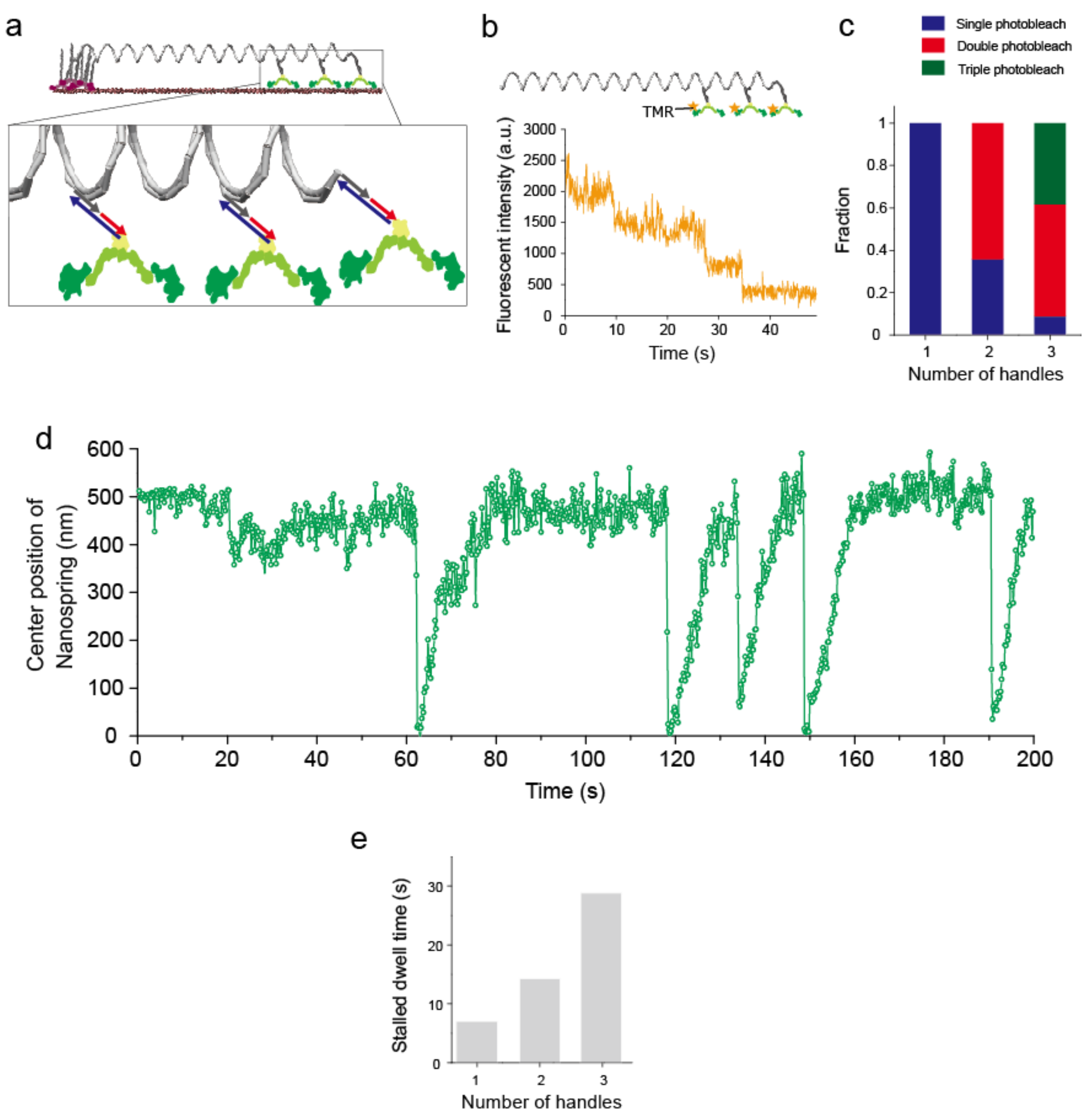

Supplementary Figure 7

Controlling the number of myosin VI attached to a nanospring.

(a) The design for attaching three myosin VIs to a nanospring. The nanospring forms a coil structure with 13.3 turns, and handles (grey arrows, **Supplementary Data**) project out at one end and 1.3 and 3.3 turns from the end. Myosin VI is dimerized by 42-mer (blue arrows) and 21-mer (red arrows) oligos and captures a handle via the 42-mer oligo.

The other end of the nanospring was attached to five handles captured by immobile myosin II to ensure the anchoring of three myosin VIs. **(b)** Photobleaching of tetramethyl-rhodamine (TMR) labeled to a myosin head dimers attached to the nanospring. An example trajectory shows TMR photobleached in three steps, suggesting three myosin VIs were attached to the nanospring. **(c)** Statistics of the photobleaching steps for nanospring designs with one, two or three handles. The results indicate on average 1, 1.6 and 2.3 myosin VIs were attached to the nanospring, respectively. However, these values are likely underestimates due to the labeling efficiency of TMR and inactivation of the dye molecules. **(d)** Fluorescently-labeled nanospring dynamics forced by myosin VIs. The center position of the fluorescence image was tracked by FIONA. Three handles were attached to the nanospring. Nucleotide condition, 2 mM ATP+100 μM ADP. Recording rate, 5 Hz. **(e)** The effect of the number of myosin VI molecules on stalled dwell time. The median values for nanosprings with one handle, two handles and three handles are 6.8 s, 14.3 s and 28.8 s, respectively. Stalled dwell time was prolonged as expected with increasing myosins, and nanosprings with three handles were frequently anchored for a minute.

Supplementary References

1. Douglas, S.M. *et al.* Rapid prototyping of 3D DNA-origami shapes with caDNAno. *Nucleic Acids Res* **37**, 5001-5006 (2009).
2. Dietz, H., Douglas, S.M. & Shih, W.M. Folding DNA into twisted and curved nanoscale shapes. *Science* **325**, 725-730 (2009).
3. Douglas, S.M. *et al.* Self-assembly of DNA into nanoscale three-dimensional shapes. *Nature* **459**, 414-418 (2009).
4. Ishijima, A. *et al.* Simultaneous observation of individual ATPase and mechanical events by a single myosin molecule during interaction with actin. *Cell* **92**, 161-171 (1998).
5. Lang, M.J., Fordyce, P.M., Engh, A.M., Neuman, K.C. & Block, S.M. Simultaneous, coincident optical trapping and single-molecule fluorescence. *Nat Methods* **1**, 133-139 (2004).
6. Hohng, S. *et al.* Fluorescence-force spectroscopy maps two-dimensional reaction landscape of the holliday junction. *Science* **318**, 279-283 (2007).
7. Brau, R.R., Tarsa, P.B., Ferrer, J.M., Lee, P. & Lang, M.J. Interlaced optical force-fluorescence measurements for single molecule biophysics. *Biophys J* **91**, 1069-1077 (2006).
8. Jauffred, L., Richardson, A.C. & Oddershede, L.B. Three-dimensional optical control of individual quantum dots. *Nano Lett* **8**, 3376-3380 (2008).
9. Iwaki, M., Iwane, A.H., Ikezaki, K. & Yanagida, T. Local heat activation of single myosins based on optical trapping of gold nanoparticles. *Nano Lett* **15**, 2456-2461 (2015).
